# Examining illness perceptions over time: an exploratory prospective analysis of causal attributions in individuals with depressive symptoms

**DOI:** 10.1186/s12888-024-05949-z

**Published:** 2024-07-16

**Authors:** Anna Katharina Reinhold, Patrick Trudzik, Anna Levke Brütt

**Affiliations:** 1 Department of Health Services Research, Faculty of Medicine and Health Sciences, Carl von Ossietzky University Oldenburg, Oldenburg, Germany; 2 Department of Medical Psychology, University Medical Center Hamburg-Eppendorf, Hamburg, Germany

**Keywords:** Depression, Illness perceptions, Causal beliefs, Causal attributions, Time-stable causes

## Abstract

**Background:**

According to the Common-Sense Model of Illness Representations, illness beliefs, such as causal attributions, can influence the way people assess and cope with their illness and vice versa. To date, causal attributions in people with depressive symptoms have been studied mainly cross-sectionally, quantitatively and independently. The purpose of this study is to examine the causal attributions of people with depressive symptoms in terms of their stability over time, dependence on treatment experience, and differentiation of causal concepts.

**Methods:**

In a population-based prospective sample, people with at least mild depressive symptoms (PHQ-9 Score ≥ 5) were interviewed via telephone at T0 and twelve months later (T1). Causal attributions were assessed using the Brief Illness Perception Questionnaire. After the open responses were qualitatively analysed using a deductive-inductive approach, stability over time was assessed for causal attributions and concepts by comparing answers between the two time points. Subsequent exploratory quantitative analyses were conducted using chi-square tests, t-tests, and logistic regression analyses.

**Results:**

A total of 471 individuals (age M = 53.9, 53.6% female) with a mean PHQ-9 Score of 8.4 were included in the analyses. Causal attributions related to participants’ social environment, workplace, and past are the most stable over time. However, individuals with and without a time-stable causal concept showed no differences in terms of sociodemographic characteristics, severity of depressive symptoms, risk of comorbidity, and treatment experiences. Overall, the causal concepts of people with depressive symptoms appear to be very diverse. Those with treatment experience (M = 2.21, SD = 0.80) named significantly more causal attributions compared to people without treatment experience (M = 1.98, SD = 0.81, t(471) = -3.060, *p* < 0.01). In addition, logistic regression analyses revealed that treatment-experienced respondents were more likely to attribute “childhood/youth/parental home” and “predisposition”.

**Conclusions:**

Our study reveals that people with treatment experience tend to report treatment-congruent causal attributions, such as childhood and family environment, as well as predisposition, more frequently. Understanding how causal attributions and concepts are formed and change can be helpful for addressing causal attributions in treatment. Future studies should take into account the benefits of employing qualitative survey methods for exploring causal attributions.

**Supplementary Information:**

The online version contains supplementary material available at 10.1186/s12888-024-05949-z.

## Background

In high-income countries such as the United States, the United Kingdom, Canada, or Germany, adequate treatment options for people with depression are available [1] and defined by established guidelines [2–5]. Nevertheless, the global 12-month treatment rate for patients with depressive disorders is estimated to be 48.3% in high-income countries [6]. Given the high prevalence (4.4% worldwide, ranging from 2.6 to 5.9% per region [7]) and burden [8, 9] of the disease, this results in a relevant treatment gap.

Health services use as a facet of health behaviour can be incorporated in the Common-Sense Model of Illness Representations (CSM) [10]. The CSM assumes that cognitive and emotional representations of illnesses can influence the way people assess and cope with their physical or mental illness. The cognitive dimension of illness representations consists of five components: cause, controllability, consequences, identity and timeline [11]. According to the CSM, illness perceptions may change over time in the process of evaluating the chosen coping strategy. As a result, the individual may alter existing illness representations, based on which future coping responses will be selected [10]. This paper focuses on the component that comprises the individual’s attribution of the aetiology of the illness (cause) as its content. Based on the attribution theory [12] some assumptions on the stability of causes can be drawn: In the case of a stable cause, individuals may expect a similar outcome, e.g., that the utilised coping strategy will be helpful again. Furthermore, it can be assumed that individuals with time-stable causal attributions have thoroughly examined their illness and its causes. Consequently, it can be concluded that they are more likely to recognize their symptoms as needing treatment and, as a result, are more likely to seek the necessary help. Stability over time can be examined in relation to individual causal attributions or to causal concepts, with the latter describing causal attributions that occur together.

Quantitative methods have been the prevalent approach for gathering causal attributions of persons with depressive symptoms in previous studies [13–18]. Typically, each cause is presented separately, with participants rating their agreement level using a five-point Likert scale. Querying the relevance of given causal attributions on a Likert scale could result in acquiescence of respondents [19], even though those reasons hold no personal significance to their own disease (i.e., „I heard of this before – this might be a reason.”). This has to be taken into account when interpreting results. In contrast, using an open-ended question to query the most significant causal attributions provides a cause generated by the individual (i.e., “This reason is meaningful for my own disease.”). Previous research has shown that the two approaches yield different results regarding causal attributions in people with depressive symptoms/disorders. For instance, biological causal attributions are cited much more frequently as a possible reason when the causal attributions are assessed quantitatively (e.g. [14–16]), compared to qualitative approaches (e.g., [20, 21]). Consent to or mention of personal or psychosocial causal attributions are found to a comparable extent across both methods [21, 22].

Using quantitative approaches to examine causal attributions, existing studies have shown that individuals’ causal beliefs about depression are multifaceted and culturally determined [23]. They are related to early steps in the help-seeking process, such as perceived need and help-seeking intention [16], treatment preferences in adult psychotherapy clients [17], treatment assignment [15], lay help-seeking beliefs [13, 18], and lay publics’ formal and informal help-seeking recommendations [14]. The results indicate that causal attributions can either hinder or facilitate the likelihood of utilising mental health care services.

The potential change in causal attributions following the utilisation of mental health care services has not been adequately studied, even though this is an integral part of the CSM [10]. Some studies have cross-sectionally assessed the influence of prior treatment experiences on causal attributions. It has been shown that increased treatment experience with psychotherapy or with medication therapy predicted higher levels of characterological and biological causal attributions [24]. A very similar relationship was found by Schweizer and colleagues [15], such that patients who had previously received some sort of treatment for their current episode of depression were more likely endorsing biological and characterological causal beliefs.

To the best of our knowledge, there is only one longitudinal study that has examined the relationship between prior treatment experiences and causal attributions among people with depressive symptoms, and provided insight into stability of causal attributions over time. Leykin and colleagues [25] assumed, based on existing evidence, that causal attributions of people with depressive disorders are associated with specific types of treatment, namely characterological causal beliefs with cognitive therapy and biological causal beliefs with antidepressant medication. Results obtained in this randomized controlled trial showed that treatment-congruent attributions of cause did not increase after treatment had occurred, while treatment-incongruent attributions of cause were less frequently reported by participants, indicating that certain treatment experiences influence causal attributions to varying degrees. This study provides initial evidence that previous treatment experience can affect causal attributions [25]. However, the concept is worth to consider because, according to the attribution theory [12], treatment-congruent attributions may have an impact on future help-seeking efforts of individuals suffering from depressive symptoms.

Besides considering causal attributions independently, it might be worthwhile to study them in concepts, namely, in conjunction with the other causal attributions mentioned by the individual. Based on previous research [23], it can be assumed that the causal concepts of people with depressive symptoms are complex. Studies that interpret the impact of each causal attribution independently [13, 16], neglect that those affected largely attribute their depressive symptoms to multiple, contrastive causes (e.g., biological reasons vs. psychosocial reasons) [21, 26]. By using a complexity score, Khalsa and colleagues [24] showed that a higher count of prior medication courses correlated with elevated complexity scores.

To the best of our knowledge, no previous study has investigated the stability of causal concepts over time. Previous studies have shown that causal attributions, when considered separately, may depend on the culture and diagnosis of the respondents [23]. Since our study is exploratory in nature, we decided to examine whether people with and without a time-stable causal concept differ in terms of the severity of their depressive symptoms, their socioeconomic characteristics, and the presence or absence of comorbidities, to detect possible differences and inform future studies.

It becomes evident that causal attributions have an impact on several stages of the help-seeking process. However, knowledge about possible changes in them during the evaluation of applied coping strategies is lacking. As stated, causal attributions have tended to be examined individually, quantitatively, and cross-sectionally. This paper aims to investigate qualitatively collected causal attributions with regard to their stability over time, the dependence on treatment experiences and the differentiation of causal concepts.

We contribute to the existing literature by first investigating the applicability of the category system established by Magaard and colleagues for clinical patients to population-based data in individuals with depressive symptoms. We will further answer the following research questions:

1. Are some causal attributions more stable over time than others?
2. Do participants with and without time-stable casual concepts differ with respect to prior specific professional and psychopharmacological treatment experiences, risk of comorbidity, their severity of depressive symptoms, and sociodemographic characteristics?
3. Does the use of mental health care services lead to certain causal attributions being mentioned more than others, as indicated in the CSM?
4. Are specific causal attributions commonly co-mentioned, resulting in distinct causal concepts?
5. Does the level of differentiation in causal concepts correlate with prior treatment experiences?

## Methods

### Recruitment and participants

The University of Oldenburg research team (UORT) commissioned the independent social research institute USUMA GmbH to recruit the participants and to interview them within the framework of two computer-assisted telephone interviews. This was part of a nationwide study titled “Influence of established and subjectively perceived as well as evaluated individual characteristics on the utilisation of mental health services among individuals with depressive disorders,” funded by the German Research Foundation (DFG) [27]. A total of 58 and 4 interviewers were deployed at T0 and T1, respectively. According to the Association of German Market and Social Research Agencies dual-frame approach the sample was generated from landline and mobile numbers [28].

Recruitment and interviews for the first study period (T0) took place from April till November 2020. Inclusion criteria were an age of 18 years or older and the presence of at least mild depressive symptoms in participants, operationalised by a score of at least five in the Patient Health Questionnaire (PHQ-9) [29]. For interviewees who did not meet the inclusion criterion (PHQ-9 score < 5, *n* = 4577), the interview ended at this point. A total of 925 participants met the inclusion criteria at T0 and agreed to participate after giving informed consent. One year after their first interview (T1), 531 participants could be contacted and interviewed again. The dropout rate was 42.6%.

Individuals who did not take part at T1 (*n* = 393), made use of their right to withdraw their consent (*n* = 1), and those who did not provide information on treatment experience (*n* = 1) or causal attributions in relation to the development of their depressive symptoms (*n* = 59 at T0 and/or T1) were excluded from the analysis. Accordingly, the analysis data set included 471 participants.

The study was approved by the Ethics Committee of the Oldenburg Medical School (ID: 2018-090). Participants scoring high on the suicidal ideation on the PHQ-9 (all other response options on the 4-point Likert scale than “not at all”) were given possible contact points to turn to in order to receive necessary help. More details on the survey method can be found in the study protocol [27]. To enhance motivation for participation at T0, USUMA GmbH, in consultation with the UORT, decided to introduce a fixed donation of 1000 Euros to the Foundation for Mental Health [30] during the course of the survey. The donation was independent of the number of participants and was approved by the ethics committee.

### Measures

Only measures that are relevant to the research questions addressed in this paper will be explained in the following (for further information see Additional file 1: Table S1). The full list of instruments can be found in the study protocol [27].

Causal attributions were surveyed qualitatively using the last item of the Brief Illness Perception Questionnaire (IPQ-Brief) [31]. This short version of the Illness Perception Questionnaire-R (IPQ-R) [32] includes nine items (selected from the psychometrically tested German translation of the IPQ-R [33]), of which the ninth item is an open-ended question that asks respondents to name the three main reasons they believe caused their illness. The term “illness” was replaced by the term “psychological complaints” since there had been no reliable depression diagnosis of the respondents at the time of the survey. If necessary, respondents were asked to summarize each causal attribution with a maximum of five keywords.

Utilisation of different providers from three areas of mental health care (outpatient care, inpatient care, low-threshold care), as well as the utilisation of prescription medication for psychological complaints were queried according to the procedure in a modular study on mental health conducted in the German adult population [34]. For the analyses in this study, only specific billable health insurance services were considered, reflecting professional and psychopharmacological treatment experiences. Accordingly, the following inpatient and outpatient facilities were included: psychiatric, psychotherapeutic or psychosomatic clinics, departments, day-care hospitals and outpatient clinics, psychosomatic rehabilitation, established psychotherapists and psychiatrists/neurologists, general practitioners (for psychological problems), and (social) psychiatric service. In addition to the inpatient and outpatient mental health services, utilisation of prescription medication for psychological complaints were considered a treatment experience, as it is indispensable to first consult a psychiatrist or general practitioner in order to receive medication. Lifetime utilisation, 12-month utilisation, and current utilisation of the various mental health care services were recorded at T0, and 12-month utilisation and current utilisation were recorded at T1.

Depression scores were collected at both time points with the PHQ-9 [29, 35], with each item corresponding to one of the nine DSM-IV criteria for diagnosing major depression. Participants rated their agreement with each item (e.g., “Little interest or pleasure in doing things”) on a four-point Likert scale ranging from 0 (“not at all”) to 3 (“nearly every day”). The resulting score can range from 0 to 27 and indicates the severity of depressive symptoms experienced over the past two weeks. The internal consistency of the questionnaire is acceptable at T1, with Cronbach’s alpha for depressive symptomatology = 0.77, slightly below that reported in validation studies (Cronbach’s alpha = 0.89 [36]) but still within an acceptable range.

Subjects were screened for the three comorbidities generalized anxiety disorder (Generalized Anxiety Disorder Scale-7 [37, 38], alcohol use disorder (Alcohol Use Disorders Identification Test - Consumption [39–41], and somatic symptom disorder (Patient Health Questionnaire 15 [29, 35] and Somatic Symptom Disorder Scale-12 [42]). Risk for comorbidity was present if at least one screening scored positive. Using the expectation maximization algorithm [43], missing values from the screening instruments were replaced if no more than 25% of the values per subject and instrument were missing. Missing values per item ranged from 0.2 to 7.4%.

The socioeconomic status index (SES) was calculated based on the data on education, job situation and net equivalence income (computed from the size of household and income/financial situation of the respondent) [44]. If missing data occurred in one of the three individual dimensions, we imputed the mean score of the remaining two dimensions. Missing data in more than one dimension meant that the SES index could not be calculated and was therefore missing. Further sociodemographic characteristics reported are age, sex, family status, and migration background (self or at least one parent not born with German citizenship) [45].

### Data analyses

#### Qualitative analysis

The IPQ-Brief open-ended answers from the T0 data set were deductively assigned to 11 existing categories [21] by two researchers (AKR and PT). These categories include problems at work, problems in social environment, self/internal states, unspecific stress and overload, negative life events, childhood/youth/parental home, physical complaints and illnesses, predisposition, social situation, fate, and insufficient treatment. In this first step, answers that could not be assigned to any of the given categories were marked and then it was discussed which categories had to be newly formed in order to be able to assign all answers. After completion of the category system, the remaining codes were assigned by both researchers and then intercoder reliability was calculated. Disagreements regarding the coding of causal attributions were discussed by AKR and PT until consensus was reached. In case of doubt, a third researcher (ALB) was included in the discussion. The T1 dataset was coded by two researchers (AKR and PT) with the revised category system. In cases respondents stated more than three causal attributions, up to five were coded. In contrast to the approach of Magaard et al. [21], we decided to code stress as specifically as possible. Therefore, stress in more than one part of life was coded twice or even three times and assigned to the corresponding category instead of combining all given answers within the category “unspecific stress and overload”. The qualitative analysis was performed with MAXQDA 2020 [46].

The existing deductive category system (*n* = 11 categories) could be applied and extended according to the methodological approach. Three new categories were formed, the first of which covers aging. This category includes causal attributions concerning the topics age-related physical complaints, fear of aging and death, retirement, loss of independence/dependence on third parties, and unspecific statements concerning old age. Because our survey coincided with the Covid-19 pandemic, we created an additional category for Covid-19 related causal attributions that included not further specified responses and responses that could be assigned to the following five themes: pandemic-related anxiety, home-schooling and home office, leisure and freedom restrictions, economic consequences due to the pandemic, and social isolation due to the pandemic. Finally, the category “environmental conditions” was newly formed. This category includes all causal attributions related to weather in general and in particular to the seasonal effects of weather in the dark season as well as noise pollution.

On the basis of this category system, the numbers of coded causal attributions were 1149 and 1121 at T0 and T1, respectively (multiple responses of the same causal attribution considered). At T0, nine reasons could not be coded, of which five reasons were not readable and four reasons could not be interpreted (e.g., " have woken up”). The intercoder reliability at T0 and T1 was 79% and 71%, respectively.

#### Quantitative analyses

To conduct quantitative analyses, the coded data were imported into SPSS. For the descriptive analysis of the first research question, a time-stable causal concept was present if the respondent mentioned the exact same casual attributions (x) at T1 compared to T0. We also defined a causal concept as time-stable if the respondent mentioned x + 1 (when stating only one causal attribution at T0) or x ± 1 (when stating more than one causal attribution at T0) identical causal attributions at T1. This liberal definition was chosen because some causal attributions are inherently more stable over time than others.

Utilisation was differentiated with regard to lifetime treatment experience before T0 (lifetime utilisation), continuous treatment experience from T0 until T1 (continuous 12-month utilisation), and new treatment experience between T0 and T1 (new 12-month utilisation). The reference group is composed of participants who have not received treatment at any time point.

To explore possible associations between the presence/absence of time-stable causal concepts and categorical as well as metric variables, chi-square tests and t-tests were calculated, respectively.

To examine possible associations between mentioning a reason at T1 and prior utilisation of specific mental health care services, chi-square tests were first calculated. Subsequently, logistic regression analyses with conditional backward selection were calculated for all significant correlations (*p* ≤ 0.05). Considered covariates were gender, severity of depressive symptoms (PHQ-9 Score at T0) and the presence of possible comorbidities.

The analysis to identify causal concepts was performed using the “simple code configuration” function in MAXQDA 2020. This can be used to display the percentage frequency of all code combinations that occurred. Since the differentiation of concepts was the focus of this analysis, multiple mentions of the same causal attribution were not considered here. Means of mentioned causal attributions between participants with and without specific professional and/or psychopharmacological treatment experience were compared using a t-test.

## Results

### Sample characteristics

Table 1 displays sample characteristics for the whole dataset and by stability of causal concepts over time. Due to missing values, the data set for this analysis was reduced to *n* = 463 subjects. The respondents were on average 53.9 years old and 53.6% were female. Majority of respondents had a high (46.2%) or middle (44.5%) socioeconomic status and approximately one-fifth had a migration background. One third of the respondents were either single or married, and the remaining were divorced (16.4%) or widowed (14.9%). The mean PHQ-9 score was 8.4 at T0, which corresponds to mild depressive symptoms. Slightly more than half of the respondents had sought specific professional and/or psychopharmacological help for their mental health symptoms at some point in their lives (54.0%). Participants who screened positive for at least one of the three surveyed comorbidities comprised 52.1%.

**Table 1:** Characteristics of the study sample by stability of causal concepts over time

	All (*n* = 463)	Time-stable causal concept^a^ (*n* = 271)	No time-stable causal concept^a^ (*n* = 192)
Sex, n (%)			
male	215 (46.4)	121 (44.6)	94 (49.0)
female	248 (53.6)	150 (55.4)	98 (51.0)
Age in years, mean (SD; min, max)	53.9 (17.5; 18, 93)	54,1 (17.2; 18, 93)	53,6 (18.0; 18, 91)
Socio economic status index, n (%)			
low	43 (9.3)	27 (10.0)	16 (8.3)
middle	206 (44.5)	121 (44.6)	85 (44.3)
high	214 (46.2)	123 (45.4)	91 (47.4)
Migration background, n (%)			
yes	94 (20.3)	53 (19.6)	41 (21.4)
no	369 (79.7)	218 (80.4)	151 (78.6)
Family status, n (%)			
single	156 (33.7)	92 (33.9)	64 (33.3)
married	162 (35.0)	93 (34.3)	69 (35.9)
separated/divorced	76 (16.4)	44 (16.2)	32 (16.7)
widowed	69 (14.9)	42 (15.5)	27 (14.1)
PHQ-9 Score T0, mean (SD; min, max)	8.4 (3.7; 5, 25)	8.4 (3.8; 5, 25)	8.3 (3.6; 5, 22)
Treatment experience^1^, n (%)			
no	213 (46.0)	126 (46.5)	87 (45.3)
lifetime utilisation	95 (20.5)	54 (19.9)	41 (21.4)
continuous 12-month utilisation	72 (15.6)	42 (15.5)	30 (15.6)
new 12-month utilisation	83 (17.9)	49 (18.1)	34 (17.7)
Risk of comorbidity			
no	222 (47.9)	129 (47.6)	93 (48.4)
yes	241 (52.1)	142 (52.4)	99 (51.6)

^a^ Chi-square tests and t-tests revealed no significant group differences at *p* ≤ 0.05

^1^ billable mental health insurance services were considered, reflecting specific professional and psychopharmacological treatment experiences

### Causal attributions stability over time and dependence on treatment experience

The causal attributions mentioned were assigned to 14 different categories, of which “problems in the social environment” (59.7%), “negative life events” (57.0%), and “problems at work” (57.0%) were the most stable over time. In contrast, “unspecific stress and overload” (21.7%), “insufficient treatment” (16.7%), and “fate” (0.0%) occurred as the least time-stable categories (Table 2). Categories were relatively independent from each other and only weakly correlated (*r* = -0.21 to *r* = 0.23; see Additional file 2). Individuals with (*n* = 271) and without (*n* = 192) time-stable causal concepts did not differ significantly from each other in any of the characteristics examined (Table 1). A sensitivity analysis including only respondents with a PHQ-9 score greater than or equal to 10 did not yield different results (see Additional file 3: Table S2).

**Table 2:** Causal attributions stability over time in descending order

	Mentioning of causal attributions per time point		Causal attributions stability over time^a^
	T0, *n* (%)	T1, *n* (%)	T0 ➔ T1, *n* (%)
Problems in social environment	201 (42.7)	210 (44.6)	120 (59.7)
Negative life events	79 (16.8)	85 (18.0)	45 (57.0)
Problems at work	172 (36.5)	154 (32.7)	98 (57.0)
Childhood, youth, parental home	53 (11.3)	79 (16.8)	30 (56.6)
Physical complaints and illnesses	120 (25.5)	91 (19.3)	63 (52.5)
Self/internal states	141 (29.9)	137 (29.1)	70 (49.6)
Covid-19 Pandemic and related consequences	89 (18.9)	70 (14.9)	32 (36.0)
Aging	20 (4.2)	23 (4.9)	6 (30.0)
Predisposition	18 (3.8)	20 (4.2)	5 (27.8)
Environmental conditions	8 (1.7)	5 (1.1)	2 (25.0)
Social situation	32 (6.8)	30 (6.4)	7 (21.9)
Unspecific stress and overload	46 (9.8)	39 (8.3)	10 (21.7)
Insufficient treatment	6 (1.3)	8 (1.7)	1 (16.7)
Fate	2 (0.4)	3 (0.6)	0 (0.0)
**Total**	**987**	**954**	**489**
**Causal attributions per participant**	2.10	2.03	1.04

*n* = 471 participants

^a^ Number and percentage of causal attributions mentioned at T0 and again at T1

Chi-square tests revealed significant associations between treatment experience of specific professional and/or psychopharmacological mental health care services and the three causal attributions “Covid-19 pandemic and consequences” (chi-square (3) = 13.50, p = .004, n = 471), “childhood/youth/parental home” (chi-square (3) = 24.29, p < .001, n = 471), and “predisposition” (chi-square (3) = 16.03, p = .001, n = 471). Subsequent logistic regressions with conditional backward selection revealed that, at T1, the odds of naming the causal attribution “Covid-19 pandemic and consequences” was lower for participants who used mental health care services in comparison to participants without treatment experiences, regardless of the timing of treatment experience. In the regression model used to examine the causal attribution “childhood/youth/parental home”, all three time points also showed significant results compared to the reference group. However, the chance of mentioning the causal attribution at T1 was higher for persons with treatment experience at any time compared to persons without treatment experience. In the third regression model that examined the association between treatment experiences and the causal attribution “predisposition,” only two time points of utilisation became significant. Individuals who had used mental health services at some point in their lives or in the 12 months between T0 and T1 were more likely to mention the reason “predisposition” compared to individuals without treatment experiences. All three final models did not contain any of the three considered covariates (Table 3).

**Table 3:** Results of logistic regression analyses predicting selected causal attributions from treatment experiences

	Covid-19 pandemic and consequences^a^			Childhood, youth, parental home^a^			predisposition^a^		
Variables	Wald Chi Square	*p*	OR (95 CI)	Wald Chi Square	*p*	OR (95 CI)	Wald Chi Square	*p*	OR (95 CI)
Treatment experience^1^									
lifetime utilisation	3.97	0.046	0.49 (0.24, 0.99)	16.42	< 0.001	3.95 (2.03, 7.67)	6.99	0.008	8.55 (1.74, 41.97)
continuous 12-month utilisation	7.11	0.008	0.27 (0.10, 0.71)	17.17	< 0.001	4.38 (2.18, 8.81)	10.22	0.001	13.03 (2.70, 62.87)
new 12- month utilisation	5.31	0.021	0.39 (0.18, 0.87)	5.43	0.020	2.40 (1.15, 5.03)	2.25	0.134	3.98 (0.65, 24.26)
Classification of Model	85.1%			83.2%			95.7%		
Nagelkerkes R^2^	0.051			0.087			0.114		

^a^ Final model without covariates

^1^ reference category was no utilisation

### Differentiation of causal concepts

**Fig. 1:**
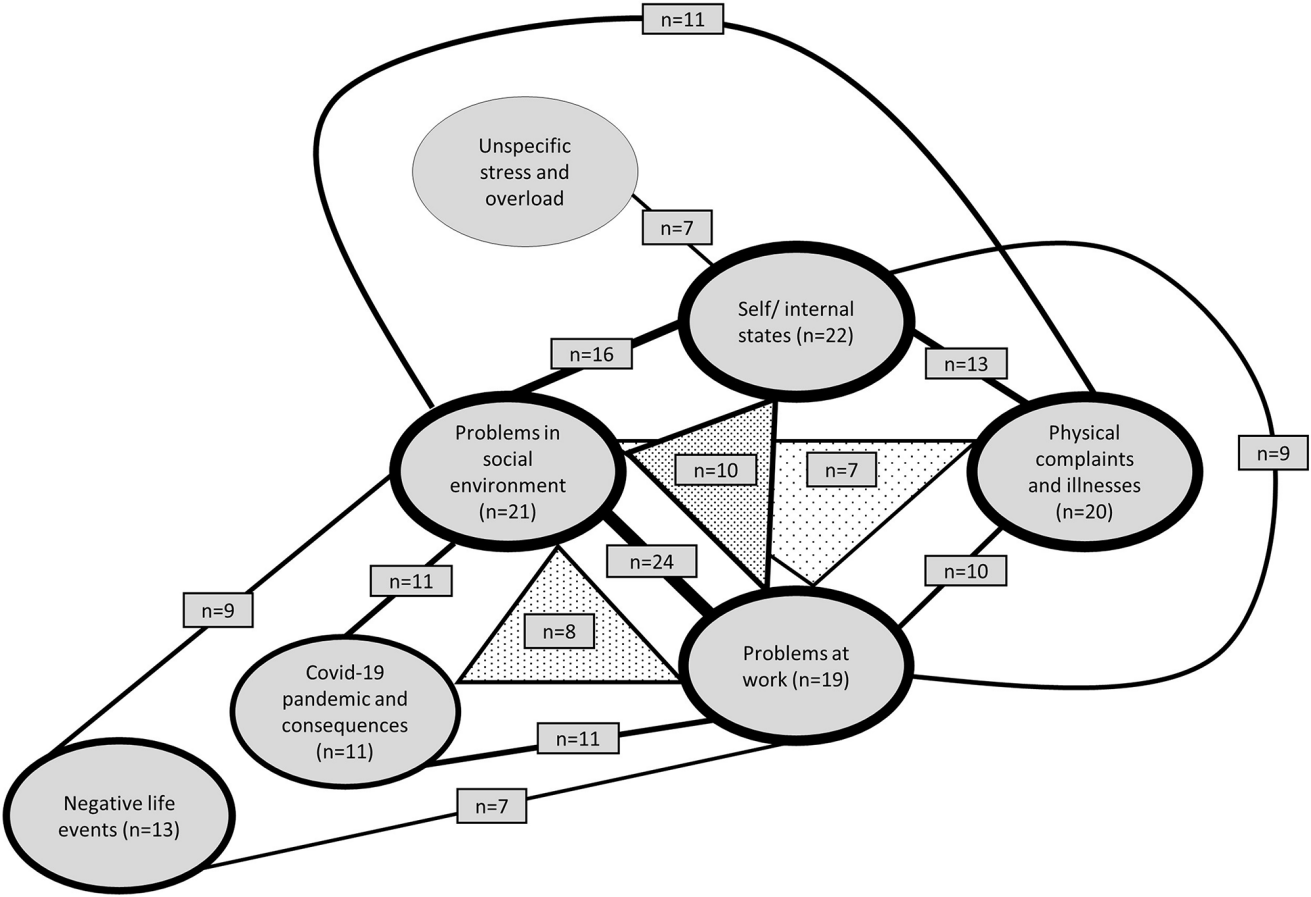
Most frequently mentioned causal attributions/concepts at T0. Frequency of included causal attributions/concepts ≥ 1.5%, *n* = 259. Thickness of the lines illustrates how often causal attributions occurred individually (thickness of the circles) or in combination (thickness of the connecting lines). Tightened triangles represent causal concepts with three distinct attributions

**Fig. 2:**
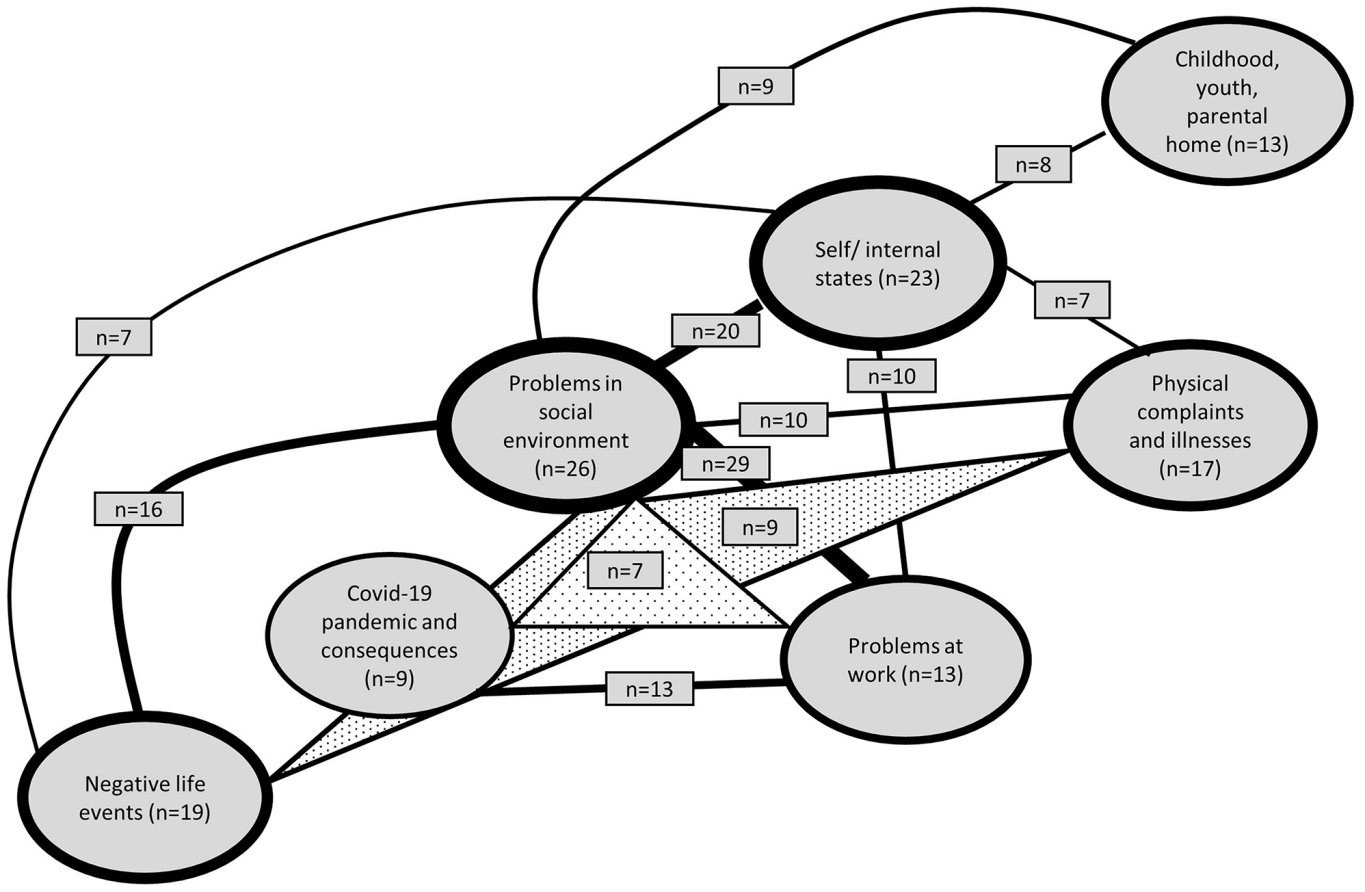
Most frequently mentioned causal attributions/concepts at T1. Frequency of included causal attributions/concepts ≥ 1.5%, *n* = 265. Thickness of the lines illustrates how often causal attributions occurred individually (thickness of the circles) or in combination (thickness of the connecting lines). Tightened triangles represent causal concepts with three distinct attributions

The causal concepts of people with depressive symptoms are diverse. At T0 and T1, 131 and 125 different concepts or single causal attributions were mentioned, respectively. The most frequently occurring causal concept at both time points comprised the two causal attributions “problems at work” and “problems in social environment” (Figs. 1 and 2). At T0, “self/internal states” was the second most mentioned causal attribution, followed by “problems in social environment” in third place. At T1, these two causal attributions occupied the second and third places in reverse order. The causal attribution “unspecific stress and overload” is no longer among the most frequent at T1, while the causal attribution “childhood/youth/parental home” only appears at T1 (Fig. 2). All causal concepts and individual causes with a minimum occurrence of seven times (equivalent to 1.5%) are displayed. It is worth noting that the composition of subjects in the two figures may differ.

If each causal attribution was considered only once (applied for analyses concerning differentiation of causal concepts), two reasons were most frequently mentioned, accounting for 43.0% at T0 (Table 4) and 45.0% at T1. Individuals with treatment experience more often reported three different causal attributions (34.6%) than individuals without treatment experience (23.0%). Overall, individuals with treatment experience mentioned more causal attributions (M = 2.21, SD = 0.80) than individuals without treatment experience (M = 1.98, SD = 0.81, t(471) = -3.060, *p* < 0.01). The effect size according to Cohen [47] is *r* = − 0.28, corresponding to a small effect.

**Table 4:** Differentiation of causal concepts at T0 by treatment experience

Number of causal attributions mentioned^1^	All (*n* = 471)	Treatment experiences (*n* = 228)	No treatment experiences (*n* = 243)
1	119 (25.3)	45 (19.7)	74 (30.5)
2	203 (43.1)	97 (42.5)	106 (43.6)
3	135 (28.7)	79 (34.6)	56 (23.0)
4	13 (2.8)	6 (2.6)	7 (2.9)
5	1 (0.2)	1 (0.4)	0
Mean (SD)*	2.10 (0.81)	2.21 (0.80)	1.98 (0.81)

^1^ multiple mentions of the same causal attribution not considered

* p-value < 0,01 based on t-test statistic

## Discussion

This prospective population-based study explored the stability of causal attributions in people with depressive symptoms over a period of twelve months. We found that problems in the participants’ social environment and workplace, as well as attributions arising from the participants’ past, are the most stable over time. In addition, we have some evidence that individuals with treatment experience had more differentiated causal concepts and named the causal attributions “childhood/youth/parental home” and “predisposition” more frequently.

We could apply the category system by Magaard and colleagues [21] at both time points and had to extend it by three categories. The occurrence of the category “environmental conditions” in our sample can possibly be explained by the inclusion of a broader, population-based sample compared to other studies [21, 24]. Correlations between, for example, weather conditions and the occurrence of depressive symptoms are known and researched [48, 49]. However, the proportion perceiving environmental conditions as causal for their depressive symptoms in our sample is rather small (1.7% at T0 and 1.1% at T1). At the beginning of the Covid-19 pandemic a significant increase in depression symptoms could have been observed worldwide [50]. Both of our survey waves coincided with the pandemic and as a result, 18.9% and 14.8% of respondents mentioned a pandemic-related causal attribution at T0 and T1, respectively, which explains the occurrence of the newly formed category “Covid-19 pandemic and consequences”. The emergence of the category “aging” can be explained by the higher proportion of people over the age of 65 in our sample (25.8% at T0 in our sample vs. 1.4% in the clinical sample (data not reported) [21]). The other causal attributions in our sample were mentioned in comparable proportions to those in the sample of Magaard and colleagues [21].

Our qualitative approach allowed us to become aware of newly emerging or temporary causal attributions. “Aging” and “Covid-19 pandemic and consequences” are in fact causal attributions that are not integrated in common questionnaires that quantitatively examine causal attributions of people with depressive symptoms [32, 51]. Consistent with previous qualitative studies [20, 21], only a very small proportion of participants in our study cited biological causal attributions compared to quantitative approaches [14, 16]. This result contrasts with the fact that biological reasons have been the most frequently investigated in past studies [23], although they may not be the most relevant for people with depressive symptoms.

We could not find any differences between participants with and without time-stable causal concepts in the characteristics we investigated, not even with regard to previous treatment experience. One possible explanation for this could be that we did not consider the individual strength of the reasons mentioned. It is possible that individuals combined less significant temporary causal attributions (e.g., the Covid-19 pandemic) with more impactful stable ones (e.g., negative life events). This could have biased the interpretation of time-stable causal concepts. Therefore, future studies should take into account the strength of these causal attributions. In the future, inquiries about causal attributions should be made more consistently and in greater depth to validly capture their temporal stability. In-person interviews may be more suitable for this purpose than telephone interviews.

However, we found that certain causal attributions seem to be more time-stable compared to others. These are “problems in social environment”, “negative life events”, and “problems at work”. It is comprehensible that these causal attributions remain more consistent over time compared to, for instance, “unspecific stress and overload”. This is because the causal attribution of “negative life events” is rooted in an enduring experience that does not fade with time, and attributions related to issues in the social environment or at work, such as relationship problems, illness of close relatives, financial problems, or problems with working conditions, also persist and do not merely become irrelevant. To date, the stability of causal attributions over time has not been considered when investigating the impact of illness perceptions on treatment decisions within the framework of the CSM [10]. In an experimental design first evidence on the influence of causal perceptions and treatment preferences was found [52]. For future research, stability over time might be a relevant factor that contributes to understanding the impact of certain causal attributions on decisions within the treatment process. For example, it would be interesting to investigate whether people who attribute time-stable causal attributions to their illness and those with time-stable causal concepts are more likely to make (repeated) use of mental health care services compared to people without time-stable causal attributions/concepts. In order to answer this question, a longitudinal survey with at least three measurement points is required.

The results of the binary logistic regression analyses revealed that treatment experienced participants were more likely to mention the causal attributions “childhood/youth/parental home” and “predisposition” and less likely to mention the causal attribution “Covid-19 pandemic and consequences” at T1 compared to treatment unexperienced participants. The two causal attributions, “childhood/youth/parental home” and “predisposition,” are already well-established and extensively researched as potential causes of depressive disorders [51]. They can be interpreted as treatment-congruent, as an objective of psychoeducation in depression treatment is to provide information on symptoms, diagnosis and causes [5]. Just like dysfunctional expectations [53, 54], treatment-incongruent causes should be stressed in treatment settings. Acknowledging predisposition as a potential cause may therefore be more likely in individuals who have undergone treatment compared to those who have not sought professional mental health care services [52]. The observation that participants without treatment experience mention the causal attribution “Covid-19 pandemic and consequences” less frequently suggests that they might experience depressive symptoms as a temporary phase triggered by the pandemic. Conversely, individuals already familiar with potential depressive symptoms are more likely to perceive the pandemic as an amplifier of their existing symptoms rather than as one of the three primary causal attributions. This hypothesis is supported by the data, indicating a significant mean difference in the PHQ-9 score between individuals who cited the pandemic as a causal attribution at T0 and those who did not (see Additional file 3: Table S3).

In our sample treatment experienced participants mentioned significantly more different causal attributions at T0 than their unexperienced counterparts. This might be due to the fact, that possible causal attributions are addressed in contact with mental health care professionals leading to more differentiated causal concepts among those in treatment. According to the CSM, social and professional influences, as well as media and public discourse, play significant roles in the formation and modification of causal beliefs about illnesses [55]. It has been shown that providing information through psychoeducational articles to untreated individuals with depression can lead to the formation of causal beliefs about the condition [52]. Our results are partially in line with the findings reported by Khalsa et al. [24]. They found that the number of prior courses of medication serve as a predictor for the endorsement of complexity scores regarding causal attributions. However, the quantity of previous psychotherapy sessions did not emerge as a predictor for endorsing complexity scores related to causal attributions [24]. Given that we evaluated specific professional treatment experience without distinguishing between types of treatment, caution is advised when interpreting the comparison. In addition, the different methodologies for collecting causal attributions (qualitative vs. quantitative) must be considered when comparing the results. The analysis revealed a multitude of distinct causal concepts, with the most prevalent concept occurring only 24 times (5.1%) at T0 and 29 times (6.2%) at T1. To date, causal attributions in people with depressive symptoms have been analysed based on single variables [15, 16]. However, in addition to ours, qualitative studies in particular have shown that people with depressive disorders consider a variety of reasons to be the cause of their illness [21, 56]. To better address this, future studies should focus on examining causal concepts rather than isolated causal attributions. Utilising methods like latent class analysis could be especially beneficial for categorizing individuals with depressive symptoms into distinct, unobserved latent classes based on their attribution of causes for their complaints.

### Limitations

While this study provides valuable insights into the time-stability of causal attributions and differentiation of causal concepts, it is essential to acknowledge its limitations. First, despite the claim to representativeness, we have a larger proportion of people with high SES in our sample than in the general population [44]. However, it cannot be presumed that this is a methodological problem, as the sampling was standardized according to the Kish-Selection-Grid technique [57]. As people with a lower SES suffer from depression more frequently than people with a higher SES [58, 59], it can be assumed that those who are severely affected were more likely to have dropped out (confirmed by dropout analysis; see Additional file 3: Table S4) or refrained from initial study participation (T0) due to their symptoms. It is further important to note that we interviewed individuals with at least mild depressive symptoms, and as a result, a definitive diagnosis cannot be presumed. However, we aimed at minimizing the number of false-negative subjects [60] by choosing a low cut-off score. This approach was in line with our objective, as we wanted to observe a risk sample over 12 months that was more likely than the general population to develop manifest depression and require appropriate help. Third, we combined treatment types into a pooled variable because our sample size was too small for detailed subgroup analyses, especially concerning medication use. Consequently, making specific statements about individual types of treatment is not feasible. Given that previous research has demonstrated the impact of diverse treatment experiences on specific causal attributions, conducting subgroup analyses would have been more advantageous. Future studies should strive for an ample number of cases to facilitate this analysis. Finally, a very large number of interviewers (*n* = 59) were required at T0 in order to realize the targeted number of cases. It is therefore possible that an interviewer bias occurred. Interviewer variability can impact the reliability and consistency of the study results. However, the usage of standardized questionnaires and a training concept for interviewers designed by the UORT can contribute significantly to reducing interviewer bias.

## Conclusions

Our results indicate noteworthy advantages of the qualitative assessment of causal attributions in individuals with depressive symptoms. These are capturing newly emerging and temporary causal factors as well as focusing on the most important causal attributions of those affected. The respondents’ causal concepts are highly diverse, with treatment experienced participants mentioning significantly more different and treatment-congruent causal attributions than their non-experienced counterparts. Understanding how causal attributions and concepts are formed and change can be helpful as they affect the help-seeking process and should be addressed in depression treatment. In our perspective, examining the stability of causal attributions over time and the differentiation of causal concepts constitutes a valuable extension to prior research in this context.

### Electronic supplementary material

Below is the link to the electronic supplementary material.

Supplementary Material 1

Supplementary Material 2

Supplementary Material 3

## Data Availability

The datasets generated and analyzed during the current study are not publicly available to protect patient confidentiality. Selected variables are available from the corresponding author upon reasonable request.

## Abbreviations

DFG: German Research Foundation
GDPR: General Data Protection Regulation
IPQ-Brief: Illness Perception Questionnaire Brief
IPQ-R: Illness Perception Questionnaire-R
PHQ-9: Patient Health Questionnaire-9
SES-Index: Socioeconomic Status Index
UORT: University of Oldenburg Research Team
