# Supplementary material for: Examining illness perceptions over time: an exploratory prospective analysis of causal attributions in individuals with depressive symptoms
Source: BMC Psychiatry. 2024 Jul 16;24:503. doi: 10.1186/s12888-024-05949-z (PMC11251109; doi:10.1186/s12888-024-05949-z)
Supplement: Supplementary file 1 — Supplementary Material 1 [file 12888_2024_5949_MOESM1_ESM.docx]

## Additional file 1

***Table S1.*** *Schedule of enrollment and assessment providing references and adaptations.*

|  | | | | Study period |  |
| --- | --- | --- | --- | --- | --- |
| Timepoint | | | | T0 | T1 |
|  | English version^a^ | German version^a^ | Adaptations | Baseline  Telephone interview | 12 months  Telephone interview |
| Variables/Measures |  |  |  |  |  |
| Age |  |  |  | X |  |
| Sex |  |  |  | X |  |
| Family status |  |  |  | X | Changes are queried |
| Migration background |  |  |  | X |  |
| Socioeconomic status | n.a. | [1]^b^ |  | X | Changes are queried |
| Depressivity (PHQ-9) | [2] | [3] | none | X ^c^ | X |
| Risk of mental comorbidity  index (GAD-7, AUDIT-C,   PHQ-15, SSD-12) | *GAD-7:* [4]  *AUDIT-C:* [5, 6]  *PHQ-15:* [7] *SSD-12:* n.a. | *GAD-7:* [8] *AUDIT-C:* [9]  *PHQ-15:* [3]  *SSD-12:* [10, 11]^b^ | none | X | X |
| Subjective illness   perception (IPQ-Brief) | [12, 13] | [14] | Since there will be no reliable diagnosis of the respondents at the time of the survey, the term "illness" will be replaced by the term "psychological complaints". | X | X |
| Mental health service use | n.a. | [15]^b^ | Response options were adapted on the basis of the pretest results (e.g., established psychotherapist instead of psychotherapist).  *Medication treatment (open-ended question)*  *Inpatient facilities:*  “Psychiatric, psychotherapeutic or psychosomatic clinic or ward”, “Psychosomatic rehabilitation”, “Psychiatric, psychotherapeutic or psychosomatic day clinic”, “Other inpatient facilities namely”  *Outpatient facilities:*  “Psychiatric, psychotherapeutic or psychosomatic outpatient clinic”, “(Socio-)psychiatric service”, “established Psychiatrist/Neurologist”, “established Psychotherapist”, “General practitioner (for mental problems)”, “Counseling centers for” (to be specified), “Other outpatient facilities namely” | X^d^ | X^e^ |

^a^ Sources for validated measurements are provided if an English version is available; for demographic measures, please see the translations below.
^b^ Available upon request in German version. ^c^ Serves as eligibility screen at T0. ^d^ Lifetime, 12-month, and point prevalence of mental health care utilisation were
assessed. ^e^ 12-month and point-prevalence were assessed with specific time spans.

| - 1. **Your sex?** | | | - 1. **Your age?** | | |
| --- | --- | --- | --- | --- | --- |
| 🞎_1_ female | | | _____ years | | |
| 🞎_2_ male  🞎_3_ divers | | |  |  |  |
| - 1. **What is your current marital status?** | | | | | |
| 🞎_1_ single | | |  | | |
| 🞎_2_ married | | |  | | |
| 🞎_3_ separated | | |  | | |
| 🞎_4_ divorced | | |  | | |
| 🞎_5_ widowed | | |  | | |
| 🞎_6_ remarried | | |  | | |
| - 1. **What is your nationality?** | | | | | |
| 🞎_1_ German | | 🞎_2_ German emigrant  (year of emigration)  Ι__Ι__Ι__Ι__Ι | | | 🞎_3_ other nationality, specifically: ________________________ |
| In which country were you born? | 🞎_1_ Germany  🞎_2_ Other country, specifically: __________________________________________ | | | | |
| In which country were your parents born? | mother:  🞎_1_ Germany  🞎_2_ Other country, specifically:  ________________________ | | | father:  🞎_1_ Germany  🞎_2_ Other country, specifically:  ____________________________ | |

## Literature

1. Lampert T, Kroll LE, Müters S, Stolzenberg H: **Messung des sozioökonomischen Status in der Studie zur Gesundheit Erwachsener in Deutschland (DEGS1)**. *Bundesgesundheitsblatt-Gesundheitsforschung-Gesundheitsschutz* 2013, **56**(5-6):631-636.

2. Kroenke K, Spitzer RL, Williams JB: **The PHQ‐9: validity of a brief depression severity measure**. *Journal of general internal medicine* 2001, **16**(9):606-613.

3. Gräfe K, Zipfel S, Herzog W, Löwe B: **Screening for psychiatric disorders with the Patient Health Questionnaire (PHQ). Results from the German validation study**. *Diagnostica* 2004, **50**(4):171-181.

4. Spitzer RL, Kroenke K, Williams JBW, Löwe B: **A Brief Measure for Assessing Generalized Anxiety Disorder: The GAD-7**. *Archives of Internal Medicine* 2006, **166**(10):1092-1097.

5. Bush K, Kivlahan DR, McDonell MB, Fihn SD, Bradley KA: **The AUDIT alcohol consumption questions (AUDIT-C): an effective brief screening test for problem drinking. Ambulatory Care Quality Improvement Project (ACQUIP). Alcohol Use Disorders Identification Test**. *Arch Intern Med* 1998, **158**(16):1789-1795.

6. Saunders JB, Aasland OG, Babor TF, De La Fuente JR, Grant M: **Development of the Alcohol Use Disorders Identification Test (AUDIT): WHO Collaborative Project on Early Detection of Persons with Harmful Alcohol Consumption-II**. *Addiction* 1993, **88**(6):791-804.

7. Kroenke K, Spitzer RL, Williams JB: **The PHQ-15: validity of a new measure for evaluating the severity of somatic symptoms**. *Psychosom Med* 2002, **64**(2):258-266.

8. Löwe B, Decker O, Müller S, Brähler E, Schellberg D, Herzog W, Herzberg PY: **Validation and standardization of the Generalized Anxiety Disorder Screener (GAD-7) in the general population**. *Med Care* 2008, **46**(3):266-274.

9. Moehring A, Rumpf H-J, Hapke U, Bischof G, John U, Meyer C: **Diagnostic performance of the Alcohol Use Disorders Identification Test (AUDIT) in detecting DSM-5 alcohol use disorders in the General population**. *Drug and Alcohol Dependence* 2019, **204**:107530.

10. Toussaint A, Löwe B, Brähler E, Jordan P: **The Somatic Symptom Disorder - B Criteria Scale (SSD-12): Factorial structure, validity and population-based norms**. *Journal of Psychosomatic Research* 2017, **97**:9-17.

11. Toussaint A, Murray AM, Voigt K, Herzog A, Gierk B, Kroenke K, Rief W, Henningsen P, Löwe B: **Development and Validation of the Somatic Symptom Disorder–B Criteria Scale (SSD-12)**. *Psychosomatic Medicine* 2016, **78**(1):5-12.

12. Broadbent E, Petrie KJ, Main J, Weinman J: **The brief illness perception questionnaire**. *Journal of psychosomatic research* 2006, **60**(6):631-637.

13. Moss-Morris R, Weinman J, Petrie K, Horne R, Cameron L, Buick D: **The Revised Illness Perception Questionnaire (IPQ-R)**. *Psychology & Health* 2002, **17**(1):1-16.

14. Glattacker M, Bengel J, Jäckel WH: **Die deutschsprachige Version des Illness Perception Questionnaire-Revised**. *Zeitschrift für Gesundheitspsychologie* 2009, **17**(4):158-169.

15. Jacobi F, Mack S, Gerschler A, Scholl L, Höfler M, Siegert J, Bürkner A, Preiss S, Spitzer K, Busch M *et al*: **The design and methods of the mental health module in the German Health Interview and Examination Survey for Adults (DEGS1‐MH)**. *International journal of methods in psychiatric research* 2013, **22**(2):83-99.
