# Supplementary material for: Examining illness perceptions over time: an exploratory prospective analysis of causal attributions in individuals with depressive symptoms
Source: BMC Psychiatry. 2024 Jul 16;24:503. doi: 10.1186/s12888-024-05949-z (PMC11251109; doi:10.1186/s12888-024-05949-z)
Supplement: Supplementary file 3 — Supplementary Material 3 [file 12888_2024_5949_MOESM3_ESM.docx]

## Additional file 3

**Table S2.** Characteristics of the study sample (PHQ-9 Score ≥ 10) by stability of causal concepts
over time.

|  | **All**  (n=135) | **Time-stable causal concept^a^** (n=80) | **No time-stable causal concept^a^** (n=55) |
| --- | --- | --- | --- |
| Sex, n (%) |  |  |  |
| male | 64 (47.4) | 36 (45.0) | 28 (50.9) |
| female | 71 (52.6) | 44 (55.0) | 27 (49.1) |
| Age in years, mean  (SD; min, max) | 53.6 (16.6; 18, 87) | 55.4 (17.7; 18, 87) | 51.0 (14.6; 18, 79) |
| Socio economic status index, n (%) |  |  |  |
| low | 17 (12.6) | 11 (13.8) | 6 (10.9) |
| middle | 68 (50.4) | 43 (53.8) | 25 (45.5) |
| high | 50 (37.0) | 26 (32.5) | 24 (43.6) |
| Migration background, n (%) |  |  |  |
| yes | 34 (25.2) | 21 (26.3) | 13 (23.6) |
| no | 101 (74.8) | 59 (73.8) | 42 (76.4) |
| Family status, n (%) |  |  |  |
| single | 49 (36.3) | 30 (37.5) | 19 (34.5) |
| married | 40 (29.6) | 19 (23.8) | 21 (38.2) |
| separated/divorced | 23 (17.0) | 13 (16.3) | 10 (18.2) |
| widowed | 23 (17.0) | 18 (22.5) | 5 (9.1) |
| PHQ-9 Score T0, mean (SD; min, max) | 13.1 (3.3; 10, 25) | 13.2 (3.5; 10, 25) | 12.9 (3.1; 10, 22) |
| Treatment experience^1^, n (%) |  |  |  |
| no | 33 (24.6) | 18 (22.8) | 15 (27.3) |
| lifetime utilisation | 24 (17.9) | 15 (19.0) | 9 (16.4) |
| continuous 12-month   utilisation | 36 (26.9) | 21 (26.6) | 15 (27.3) |
| new 12-month  utilisation | 41 (30.6) | 25 (31.6) | 16 (29.1) |
| Risk of comorbidity |  |  |  |
| no | 43 (31.9) | 29 (36.3) | 14 (25.5) |
| yes | 92 (68.1) | 51 (63.7) | 41 (74.5) |

^a^ Chi-square tests and t-tests revealed no significant group differences at p ≤ 0.05. ^1^ billable mental health insurance services were considered, reflecting specific professional and
 psychopharmacological treatment experiences.

**Table S3.** PHQ9-Score at T0 by attribution of Covid-19 Pandemic as causal for psychological complaints.

|  | **PHQ9-Score** | | **Levene's test for equality of variance** | | **t-test** | | |
| --- | --- | --- | --- | --- | --- | --- | --- |
|  | *n* | *M* (*SD*) | *F* | *Sig.* | *t* | *df* | *Sig.* |
| **Mention of Covid-19  Pandemic and related  consequences** | | | | | | | |
| yes | 89 | 7.66 (3.03) | 4.178 | 0.042* | 2.556^a^ | 162 | 0.012* |
| no | 382 | 8.63 (3.87) |  |  |  |  |  |

* p-value < 0,05 based on t-test statistic.
^a^ t-test with Welch correction for variance heterogeneity.

**Table S4.** PHQ9-Score at T0 by dropout status.

|  | **PHQ9-Score** | | **Levene's test for equality of variance** | | **t-test** | | |
| --- | --- | --- | --- | --- | --- | --- | --- |
|  | *n* | *M* (*SD*) | *F* | *Sig.* | *t* | *df* | *Sig.* |
| **Dropout after T0** | | | | | | | |
| yes | 393 | 8.85 (4.41) | 11.363 | <.001* | 2.033^a^ | 745 | 0.042* |
| no | 531 | 8.30 (3.63) |  |  |  |  |  |

* p-value < 0,05 based on t-test statistic.
^a^ t-test with Welch correction for variance heterogeneity.
